# Corrosion Behavior of Ultra-High-Strength Hot-Press-Formed B-Pillar Parts

**DOI:** 10.3390/ma19050976

**Published:** 2026-03-03

**Authors:** KyungBin Ahn, JuYeon Jin, JoungSeok Oh, HeeJin Jang

**Affiliations:** 1 Department of Materials Science and Engineering, Chosun University, 60, Chosun-dae 5-gil, Dong-gu, Gwangju 61452, Republic of Korea; 2 Department of Semiconductor Convergence, Chosun University, 60, Chosun-dae 5-gil, Dong-gu, Gwangju 61452, Republic of Korea; 3 HYUNDAI HI-TEC Industry Co., Ltd., 47, Hanamsandan 6beon-ro, Gwangsan-gu, Gwangju 62216, Republic of Korea

**Keywords:** B pillar, hot-press forming, corrosion, chloride ion, residual stress

## Abstract

The corrosion behavior of hot-press-formed (HPF) B-pillar components fabricated from Al–Si-coated boron steel was investigated with an emphasis on the forming-induced crack morphology. The specimens were extracted from the inner and outer surfaces of the top, flat, and radius regions. Microstructural characteristics and coating cracks were examined using optical microscopy, as well as field-emission scanning electron microscopy (FE-SEM) in combination with energy-dispersive spectroscopy (EDS), and corrosion behavior was evaluated using cyclic corrosion immersion and potentiodynamic polarization tests in a 3.5 wt.% NaCl aqueous solution. The Al–Si coating exhibited a multilayered structure composed of alternating Al- and Fe-rich layers. The crack morphology strongly depended on the local stress state: wide macrocracks were mainly formed on the outer surface of the radius region under tensile deformation, whereas the narrow microcracks predominated on the inner surface subjected to compressive deformation. Cyclic corrosion immersion tests showed that the corrosion propagated preferentially along the coating cracks and was more severe on the inner surfaces, where narrow microcracks promoted aggressive crevice corrosion owing to chloride ion accumulation and local acidification. By contrast, wider macrocracks on the outer surface mitigated crevice corrosion by allowing electrolyte exchange. Potentiodynamic polarization tests indicated similar corrosion rates for all regions; however, the outer radius region exhibited a relatively noble corrosion potential owing to oxide film formation on the locally exposed substrate areas. These results demonstrate that the crack morphology induced by curved forming is a key factor governing the corrosion behavior of HPF B-pillar components.

## 1. Introduction

The application of ultra-high-strength hot-press-formed (HPF) components to improve crashworthiness and reduce vehicle weight [1,2,3,4] has been steadily increasing in the automotive industry. Among these components, the B-pillar component is a critical structural member located at the center of the vehicle body, playing a key role in protecting the passenger compartment during side impacts, while also contributing to door fixation and overall body stiffness. To meet these performance requirements, tensile strengths exceeding 1.5 GPa are typically required, even with a reduced component thickness [5,6,7,8,9]. Consequently, B-pillars are commonly manufactured from boron-alloyed steel sheets using a hot-press forming process [3,10,11,12,13,14].

In the hot-press forming process, Al–Si-coated steel sheets are widely employed to suppress surface oxidation and decarburization during the high-temperature austenitization [15,16,17,18]. However, the combined effects of elevated temperatures, rapid cooling, and complex deformation histories during forming can induce coating damage, particularly in regions with large curvatures or dominant tensile deformations. In such areas, wide macrocracks frequently form within the Al–Si coating layer [19,20,21]. These cracks and defects can act as preferential pathways for electrolyte penetration in corrosive environments, thereby accelerating localized corrosion depending on the coating composition and microstructural characteristics [22,23].

Previous studies on the corrosion resistance of HPF Al–Si-coated steels have primarily been focused on flat sheet specimens, investigating the effects of heat treatment conditions, alloy composition, and microstructural evolution on the corrosion behavior [24,25]. However, actual B-pillar components exhibit a combination of complex curved geometries and flat regions, leading to non-uniform stress distributions and cooling conditions during forming [26,27,28,29]. Consequently, the coating crack morphology and substrate microstructure may vary significantly depending on the local position within the component. Nevertheless, systematic investigations, comparing the corrosion behavior of different regions in actual HPF B-pillar components, particularly curved regions, remain limited.

Therefore, in this study, the corrosion behavior of a commercial HPF B-pillar component was investigated by considering the coating crack characteristics induced by forming. The component was divided into top flat and radius regions and further classified into inner and outer surfaces. The coating and substrate microstructures were examined using optical microscopy and field-emission scanning electron microscopy (FE-SEM)/energy-dispersive spectroscopy (EDS), and residual stress measurements were conducted to correlate stress state with crack morphology. Cyclic corrosion immersion tests in a 3.5 wt.% NaCl aqueous solution were performed to analyze corrosion propagation behavior over time. Additionally, potentiodynamic polarization measurements were obtained to quantitatively compare the corrosion potential and corrosion rate of each region. Using this approach, the effects of micro- and macrocracks formed by curved forming on the corrosion resistance of HPF B-pillar components were systematically evaluated.

## 2. Experimental Procedures

### 2.1. Specimen Preparation

The B-pillar component investigated in this study was a commercial product manufactured from SABC1470 steel sheets hot-dip-coated with Al–9–11 wt.% Si, followed by the hot-press forming process. The specimens were sectioned from the B-pillar using water-jet cutting to minimize thermal and mechanical damage during preparation.

**Figure 1:**
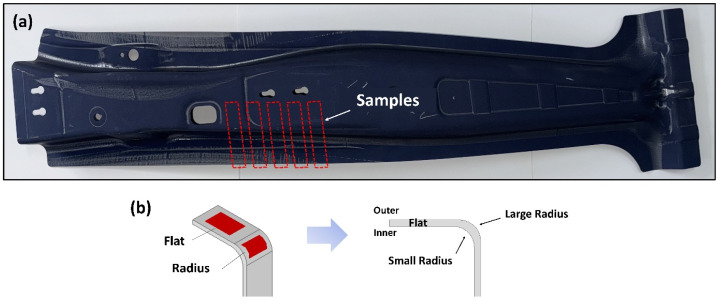
Schematic illustration of the B-pillar component showing the locations of specimens used for (**a**) cyclic corrosion immersion tests and (**b**) potentiodynamic polarization tests.

To evaluate the influence of the forming-induced geometry on the corrosion behavior, the specimen extraction locations were classified into two representative regions with different forming characteristics: the top flat region and radius region. Each region was further divided into inner and outer surfaces, corresponding to the interior and exterior sides of the vehicle, respectively. Four types of specimens were investigated in this study: inner top flat ((Inner)TF), outer top flat ((Outer)TF), inner small radius ((Inner)SR), and outer large radius ((Outer)LR). The detailed specimen locations and nomenclature used for the cyclic corrosion immersion and potentiodynamic polarization tests are summarized in Figure 1 and Table 1.

### 2.2. Microstructural and Coating Characterization

To observe the microstructure and coating morphology, the specimens were cold-mounted in epoxy resin and sequentially ground using SiC papers from #220 to #4000 grit, followed by final polishing with 1 μm alumina suspension. After polishing, the specimens were ultrasonically cleaned in ethanol and air-dried.

Cross-sectional microstructural observations were conducted using an optical microscope to examine the coating layer, interdiffusion layer between the coating and substrate, and overall substrate microstructure, with specific focus on the morphology, width, and distribution of the micro- and macrocracks formed within the Al–Si coating layer. Additionally, residual stress measurements were performed using X-ray diffraction to quantitatively evaluate the stress state in the coating and correlate the residual stress with the crack characteristics in different regions of the B-pillar. The measurements were conducted using the sin^2^ψ method with Cr-Kα radiation, and stress analysis was carried out based on the α-Fe (211) diffraction peak. The residual stresses were calculated using appropriate elastic constants, and the results represent the near-surface stress state within several micrometers of the coating/substrate interface.

Furthermore, microstructural and compositional analyses of the coating and interdiffusion layers were performed using FE-SEM (SU-8600, Hitachi, Tokyo, Japan) in combination with EDS (Hitachi, Tokyo, Japan). Elemental distribution maps were obtained to identify the Al-rich and Fe-rich phases within the coating and assess the compositional variations associated with crack formation.

### 2.3. Cyclic Corrosion Immersion Test

To evaluate corrosion behavior in chloride-containing aqueous environments, cyclic corrosion immersion tests were performed using a 3.5 wt.% NaCl aqueous solution. Each corrosion cycle consisted of immersing the specimen in the solution for 50 min, followed by a drying period of 10 min in air. During the immersion stage, the specimen surface was continuously exposed to chloride ions, allowing corrosion reactions to proceed. However, during the drying stage, corrosion products remained on the surface, promoting localized corrosion during subsequent cycles.

All the tests were conducted at room temperature (22 ± 2 °C). The total test duration was 240 cycles, and the specimens were retrieved at intervals of 48 cycles. After each retrieval, the corrosion products were removed using ultrasonic cleaning, and the specimens were cold-mounted for cross-sectional observation. The same specimen was not re-tested after cleaning; each specimen was subjected to a continuous corrosion test until its designated retrieval cycle. The corrosion morphologies and damage depths of the inner and outer surfaces of the top flat and radius regions were qualitatively compared.

### 2.4. Potentiodynamic Polarization Test

Potentiodynamic polarization tests were conducted to quantitatively compare the corrosion resistances of different regions of the B-pillar. Electrochemical tests were performed using naturally aerated 3.5 wt.% NaCl aqueous solution at room temperature using a conventional three-electrode cell. The B-pillar specimen served as the working electrode, while a Ag/AgCl electrode and a platinum wire were used as the reference and counter electrodes, respectively.

Prior to the polarization measurements, the open-circuit potential (OCP) was monitored for 30 min to stabilize the electrochemical system. Polarization tests were then conducted by scanning the potential from −0.5 V_OCP_ to 0 V_Ag/AgCl_ at a scan rate of 1 mV/s. The corrosion potential (E_corr_) and corrosion current density (i_corr_) were determined from the obtained polarization curves, and the corrosion behaviors of each region were quantitatively compared.

## 3. Results and Discussion

### 3.1. Microstructural Characteristics and Coating Crack Morphology

**Figure 2:**
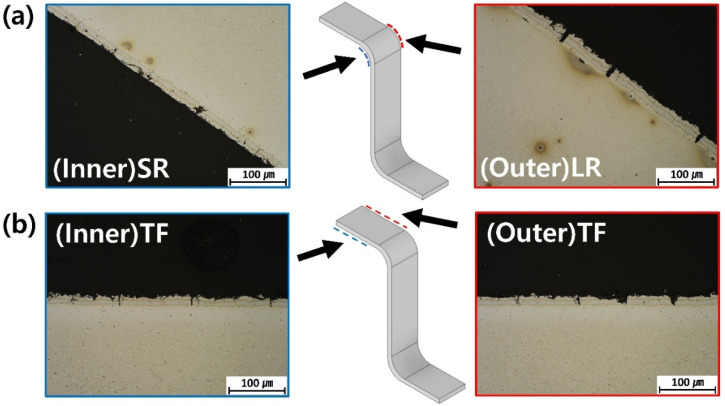
Cross-sectional microstructures of the B-pillar components observed by optical microscopy: (**a**) radius region and (**b**) top flat region.

**Figure 3:**
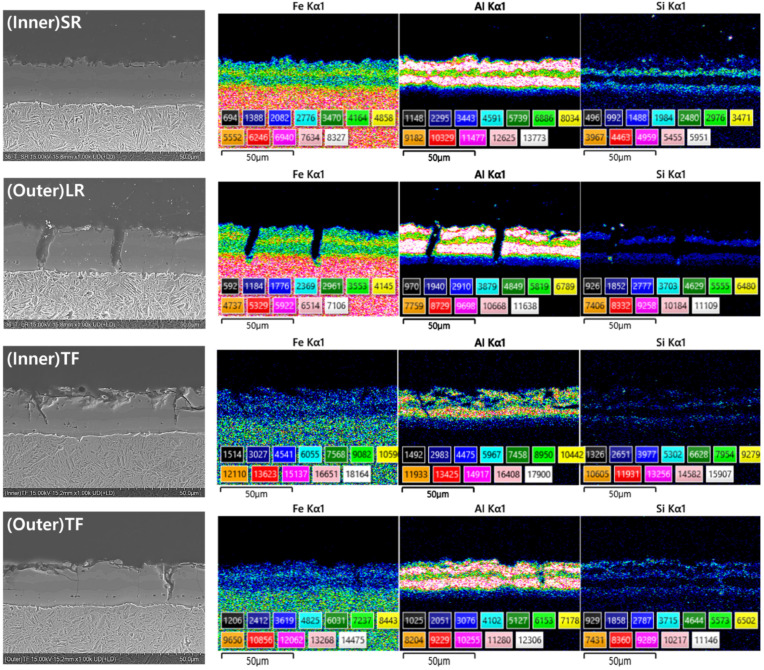
FE-SEM images and EDS elemental mapping results of the inner and outer surfaces in the radius and top flat regions of the B-pillar components.

Figure 2 and Figure 3 present the cross-sectional microstructural observations of the HPF B-pillar components obtained using optical microscopy and FE-SEM/EDS, respectively. The Al–Si coating, formed during the hot-press forming process, exhibits a characteristic multilayered structure consisting of alternating Al- and Fe-rich layers. This layered structure is attributed to the mutual interdiffusion between the steel substrate and the Al–Si coating during the austenitization stage, where Fe diffuses outward into the coating and Al diffuses inward towards the substrate. Consequently, the coating comprises a complex mixture of Al-rich and Fe-rich intermetallic layers, whereas the steel substrate transforms into a lath martensitic microstructure after rapid quenching [30].

A pronounced difference in the coating crack morphology is observed between the inner and outer surfaces, particularly in the radius region, as shown in Figure 2a. In the (Outer)LR region, numerous wide macrocracks penetrating the entire thickness of the coating are observed. These cracks typically exhibit widths of several micrometers and extend from the coating surface to the coating–substrate interface, providing direct pathways for electrolyte penetration in corrosive environments. In contrast, the (Inner)SR region predominantly exhibits narrow microcracks that are sparsely distributed and mainly confined to the coating surface or near the coating–substrate interface; continuous through-thickness cracks are rarely observed in this region.

To further quantify the observed crack morphology, crack width measurements were performed using cross-sectional OM images. The average crack width in the (Outer)LR region was 8.33 ± 0.48 µm, whereas that in the (Inner)SR region was only 1.27 ± 1.17 µm, indicating a pronounced difference in the radius region. In the top flat regions, the average crack widths were 3.85 ± 1.59 µm for (Outer)TF and 2.73 ± 0.55 µm for (Inner)TF, showing a relatively smaller difference compared to the radius region. These quantitative results confirm that the crack opening behavior is strongly influenced by the deformation mode, particularly in the radius region.

These differences in crack morphology can be directly correlated with the deformation mode during the curved forming. In the radius region, the outer surface experienced a dominant tensile deformation, whereas the inner surface was subjected to compressive deformation. Under tensile deformation, the brittle Al–Si coating was stretched, promoting crack opening and coalescence into wide, continuous macrocracks. Conversely, the compressive deformation of the inner surface suppressed crack opening, resulting in the formation of narrower and more localized microcracks. This observation clearly demonstrates that the tensile deformation during the curved forming plays a decisive role in the initiation and growth of macrocracks within the Al–Si coating.

The influence of the curvature-induced deformation is relatively limited in the top flat region compared to that in the radius region, as shown in Figure 2b. Accordingly, both (Inner)TF and (Outer)TF exhibit more uniform crack distributions, with fewer large macrocracks and a predominance of fine microcracks. Nevertheless, discontinuous cracks and coating defects were still observed, which are likely associated with the thermal and residual stresses generated during the hot-press forming process.

**Figure 4:**
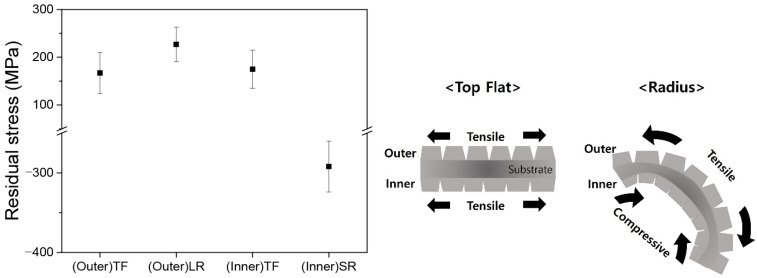
Residual stress measurement results obtained by X-ray diffraction and a schematic illustration of the residual stress distribution in the B-pillar component.

To quantitatively evaluate the stress state associated with these crack morphologies, residual stress measurements were conducted using X-ray diffraction, and the results are summarized in Figure 4. The measured residual tensile stresses are 227 ± 43 MPa for (Outer)LR, 167 ± 43 MPa for (Outer)TF, and 175 ± 40 MPa for (Inner)TF, whereas a compressive residual stress of −292 ± 32 MPa is measured for (Inner)SR. These results indicate that the B-pillar component retains a complex residual stress distribution after forming, with the (Outer)LR region experiencing the highest tensile residual stress and the (Inner)SR region dominated by compressive residual stress.

The residual stress distribution exhibits a strong correlation with the observed crack characteristics. The highest tensile residual stress measured in (Outer)LR is consistent with the prevalence of wide macrocracks in this region, suggesting that the tensile residual stress further promotes crack opening and growth. In contrast, the large compressive residual stress in (Inner)SR appears to constrain the crack opening, limiting crack growth to narrow microcracks. These findings indicate that both the forming-induced deformation and residual stress play critical roles in determining the coating crack morphology in HPF B-pillar components.

### 3.2. Corrosion Behavior During the Cyclic Corrosion Immersion Testing

**Figure 5:**
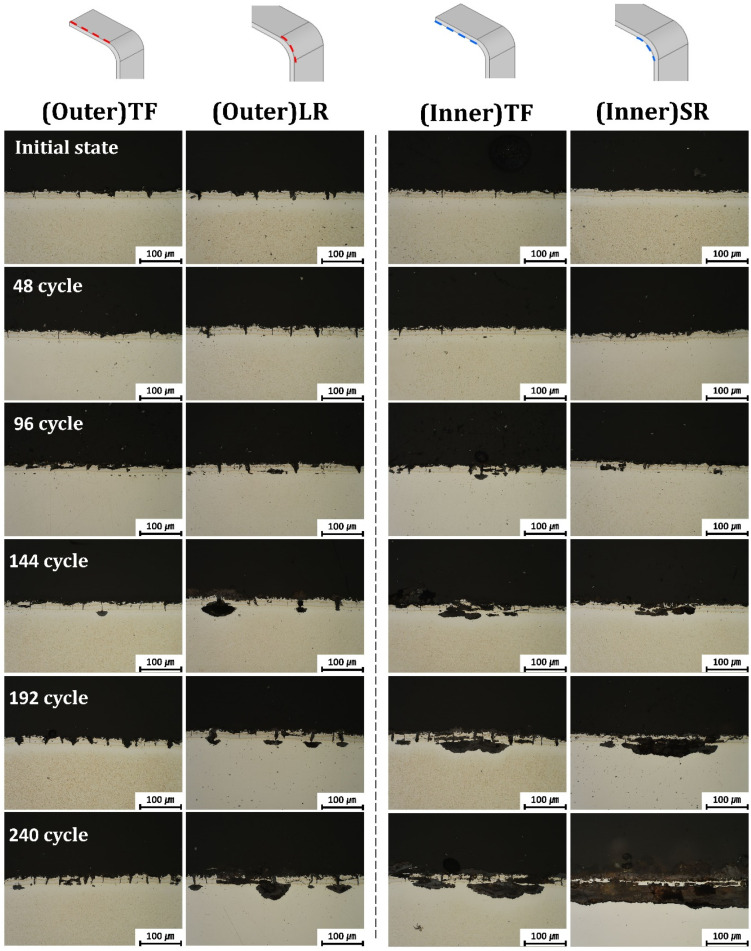
Cross-sectional microstructural evolution of the B-pillar specimens after different numbers of cycles during cyclic corrosion immersion testing.

The corrosion behavior of the B-pillar components was evaluated by cyclic corrosion immersion tests. The representative cross-sectional observations after different numbers of cycles are shown in Figure 5. After 48 cycles, minor corrosion damage was observed in all regions, which was primarily localized within the coating layer. As the number of cycles increased, corrosion preferentially propagated along the pre-existing coating cracks, gradually penetrating the coating–substrate interface and, in some cases, the steel substrate. In the advanced stages of testing, localized coating delamination and substrate exposure were observed in several regions.

Overall, the corrosion damage was more severe on the inner surfaces than on the outer surfaces, regardless of whether the region was flat or curved. The FE-SEM/EDS analysis revealed that corrosion initially proceeded through the preferential dissolution of Al-rich layers within the coating, leaving behind Fe-rich phases. This behavior is consistent with previous studies that reported the sacrificial dissolution of Al-rich phases in Al–Si coatings in chloride-containing environments, followed by accelerated corrosion at the exposed Fe-rich layers and coating–substrate interfaces [26,29,31].

A more pronounced difference in corrosion behavior was observed between the inner and outer surfaces in the radius region. In the (Inner)SR region, corrosion propagated rapidly along numerous microcracks, leading to deep localized corrosion penetrating the steel substrate. In contrast, although the corrosion propagated along the macrocracks in the (Outer)LR region, the actual damage depth and affected substrate area were limited compared to those in the inner region.

**Figure 6:**
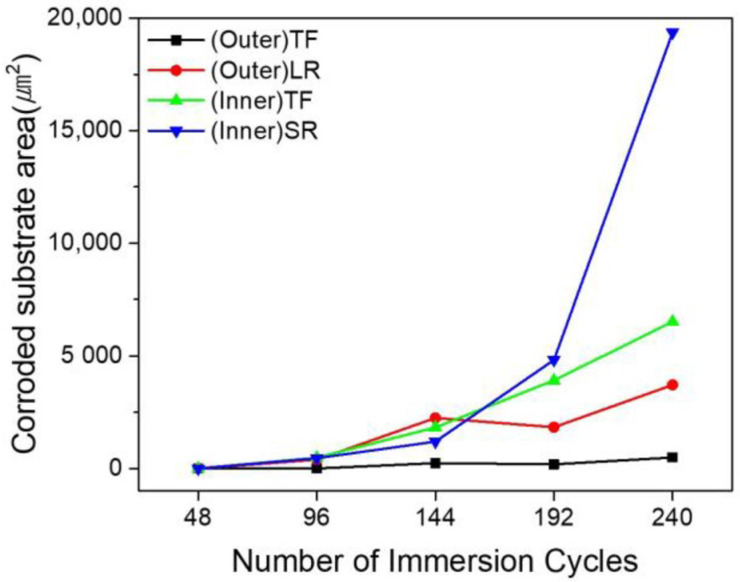
Evolution of average corrosion penetration depth with immersion cycles for the (Outer)TF, (Outer)LR, (Inner)TF, and (Inner)SR regions.

The corroded substrate area, excluding degradation confined to the Al–Si coating, was quantitatively evaluated from representative cross-sectional micrographs using image analysis, and the results are presented in Figure 6. The quantified values correspond to the localized corroded area within the analyzed cross-sectional field of view rather than the total specimen surface area. The substrate corrosion area progressively increased with increasing immersion cycles in all regions. No substrate corrosion was observed at 48 cycles; however, corrosion began to appear after 96 cycles. At 240 cycles, the (Inner)SR region exhibited the largest corroded substrate area of 19,362.63 μm^2^. This was followed by 6523.18 μm^2^ in the (Inner)TF region, 3710.97 μm^2^ in the (Outer)LR region, and 487 μm^2^ in the (Outer)TF region. These results indicate that the regional differences in substrate corrosion area became increasingly pronounced with prolonged immersion time.

This contrast can be explained by differences in the local electrochemical environments formed within the micro- and macrocracks. The narrow and partially closed, present in the (Inner)SR region, facilitate electrolyte entrapment during immersion and restrict solution exchange during the drying stage. Consequently, aggressive crevice corrosion conditions develop within these cracks, which are characterized by localized acidification and chloride ion enrichment. Such environments are known to significantly accelerate corrosion propagation, particularly in narrow crevices, where mass transport is limited [23].

By contrast, the macrocracks observed in the (Outer)LR region are wider and more open to the external environment, allowing relatively easy ingress and egress of the electrolyte during cyclic immersion. Consequently, the residence time of the electrolyte within the crack is shorter, and the extents of localized acidification and chloride accumulation are reduced compared to those in microcracks. Consequently, although the macrocracks serve as pathways for electrolyte penetration, the severity of crevice corrosion is mitigated, leading to less pronounced substrate damage.

Similar trends were observed in the top flat region. The (Inner)TF specimens exhibited more extensive corrosion damage than the (Outer)TF specimens, with corrosion penetrating deeper into the substrate along the narrow cracks. This behavior further supports the conclusion that the crack width and geometry, rather than the presence of cracks alone, are critical factors governing the severity of corrosion. Narrow cracks promote the formation of aggressive crevice environments, whereas wider cracks tend to alleviate such effects, owing to the enhanced solution exchange.

Overall, the results of the cyclic corrosion immersion tests demonstrate a strong correlation between the coating crack morphology, residual stress distribution, and corrosion behavior. The regions dominated by compressive residual stress and narrow microcracks, particularly the inner radius region, were the most susceptible to severe localized corrosion, whereas the regions with tensile residual stress and wide macrocracks exhibited relatively mitigated corrosion damage.

### 3.3. Potentiodynamic Polarization Behavior

**Figure 7:**
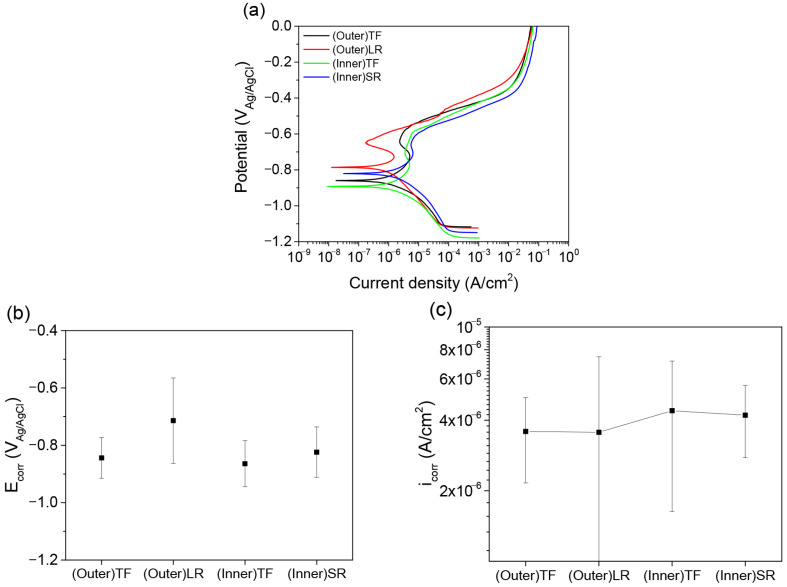
Potentiodynamic polarization test results: (**a**) polarization curves, (**b**) corrosion potential, and (**c**) corrosion current density for the B-pillar specimens.

The potentiodynamic polarization curves obtained for the top flat and radius regions on the inner and outer surfaces are shown in Figure 7. All the specimens exhibit similar general polarization behaviors, characterized by an initial active dissolution region, followed by a relatively stable passive-like region. At potentials around −0.6 V versus Ag/AgCl, a renewed increase in current density is observed, which is attributed to the breakdown of passive films associated with Fe-rich phases formed within the Al–Si coating during hot-press forming.

A comparison of the corrosion potentials revealed distinct differences between regions. In the radius region, the average corrosion potential of (Outer)LR (−0.714 V_Ag/AgCl) was significantly nobler than that of (Inner)SR (−0.824 V_Ag/AgCl). In contrast, the corrosion potentials of (Outer)TF and (Inner)TF in the top flat region were similar, measured at −0.844 V and −0.864 V_Ag/AgCl, respectively. The more pronounced difference observed in the radius region highlights the influence of crack morphology on electrochemical behavior.

The nobler corrosion potential observed in (Outer)LR is attributed to the formation of relatively stable oxide films on locally exposed substrate areas through macrocracks. Although the macrocracks allow direct exposure of the steel substrate, the oxide films formed on these exposed areas can stabilize the electrochemical response, resulting in a higher measured corrosion potential. The relatively large standard deviation of the corrosion potential reflects the variability in the crack distribution and exposed substrate area among the specimens.

The corrosion current densities of all four specimen types ranged from 3.55 × 10^−6^ to 4.39 × 10^−6^ A/cm^2^, indicating no significant difference in average corrosion rate under the short-term polarization test conditions. This suggests that, during polarization, both intact coating regions and crack-exposed regions contribute to the overall electrochemical response, limiting the sensitivity of the corrosion current density to localized damage.

**Figure 8:**
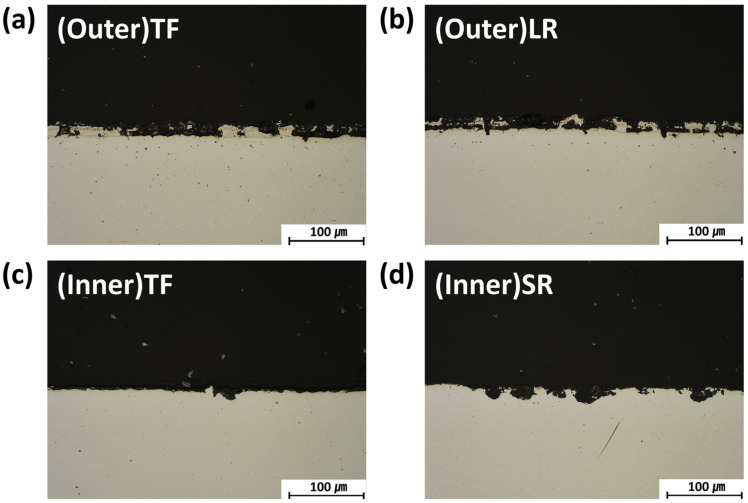
Cross-sectional optical micrographs of the B-pillar specimens after potentiodynamic polarization testing: (**a**) (Outer)TF, (**b**) (Outer)LR, (**c**) (Inner)TF, and (**d**) (Inner)SR.

The cross-sectional observations after the potentiodynamic polarization testing are shown in Figure 8. For the (Outer)TF and (Outer)LR specimens, the corrosion is largely confined to the coating layer, with preferential dissolution of Al-rich phases and limited substrate damage. In contrast, the (Inner)TF and (Inner)SR specimens exhibit extensive coating degradation, with corrosion penetrating the substrate along the microcracks. The (Inner)SR region, characterized by compressive residual stress and a high density of microcracks, shows the most severe localized corrosion at the coating–substrate interface.

The electrochemical results indicate that the (Outer)LR region exhibits the most favorable corrosion performance, despite the presence of macrocracks. The wide and open nature of these cracks suppresses the formation of aggressive crevice environments, whereas oxide film formation on the exposed substrate areas contributes to a nobler corrosion potential. These trends are consistent with the results of the cyclic corrosion immersion tests, reinforcing the conclusion that the crack geometry and openness, rather than the presence of cracks alone, govern the corrosion resistance of HPF B-pillar components.

## 4. Conclusions

The corrosion behavior of HPF B-pillar components was investigated with respect to the local microstructure and coating crack characteristics, using cyclic corrosion immersion and potentiodynamic polarization tests. The main conclusions are summarized as follows:

1. The Al–Si coating exhibited a multilayered structure consisting of alternating Al- and Fe-rich layers, and the crack morphology was strongly dependent on the forming-induced stress state. Tensile deformation in the (Outer)LR region resulted in wide macrocracks, whereas compressive deformation in the (Inner)SR region resulted in the formation of narrow microcracks.
2. Cyclic corrosion immersion tests revealed that the corrosion preferentially propagated along the coating cracks and was more severe on the inner surfaces than on the outer surfaces. The enhanced corrosion on the inner surface was attributed to crevice corrosion occurring within narrow microcracks, where chloride ion accumulation and local acidification accelerated corrosion, whereas wider macrocracks on the outer surface mitigated aggressive crevice conditions owing to enhanced electrolyte exchange.
3. Potentiodynamic polarization tests showed similar corrosion rates for all regions; however, the (Outer)LR region exhibited a relatively noble corrosion potential, owing to oxide film formation on the locally exposed substrate areas and the suppression of crevice-corrosion-prone environments.

Overall, the results demonstrate that the inner regions containing microcracks are the most susceptible to corrosion, and that the crack morphology induced by curved forming is a key factor governing the corrosion behavior of HPF B-pillar components.

## Figures and Tables

**Table 1:** Specimen nomenclature for potentiodynamic polarization tests.

	Top Flat	Small Radius	Large Radius
**Inner**	(Inner)TF	(Inner)SR	-
**Outer**	(Outer)TF	-	(Outer)LR

## Data Availability

The original contributions presented in this study are included in the article. Further inquiries can be directed to the corresponding author.
